# The Hepatic Innovation Team Collaborative: A Successful Population-Based Approach to Hepatocellular Carcinoma Surveillance

**DOI:** 10.3390/cancers13092251

**Published:** 2021-05-07

**Authors:** Shari S. Rogal, Vera Yakovchenko, Rachel Gonzalez, Angela Park, Lauren A. Beste, Karine Rozenberg-Ben-Dror, Jasmohan S. Bajaj, Dawn Scott, Heather McCurdy, Emily Comstock, Michael Sidorovic, Sandra Gibson, Carolyn Lamorte, Anna Nobbe, Maggie Chartier, David Ross, Jason A. Dominitz, Timothy R. Morgan

**Affiliations:** 1 Center for Health Equity Research and Promotion, VA Pittsburgh Healthcare System, University Drive (151C), Pittsburgh, PA 15240, USA; sandra.gibson2@va.gov (S.G.); carolyn.lamorte@va.gov (C.L.); 2 Departments of Medicine and Surgery, University of Pittsburgh, 3550 Terrace Street, Pittsburgh, PA 15240, USA; 3 Center for Healthcare Organization and Implementation Research, VA Bedford Healthcare System, 200 Springs Road (152), Building 70, Bedford, MA 01730, USA; vera.yakovchenko@va.gov; 4 Department of Veterans Affairs, Sierra Pacific Veterans Integrated Service Network, Pharmacy Benefits Management, Mather, CA 94523, USA; rachel.gonzalez@va.gov; 5 Office of Healthcare Transformation, Department of Veterans Affairs, 810 Vermont Avenue, Washington, DC 20420, USA; angela.park@va.gov; 6 Division of General Internal Medicine, Department of Medicine, University of Washington School of Medicine, 1959 NE Pacific Street, Seattle, WA 98195, USA; lauren.beste@va.gov; 7 General Medicine Service, VA Puget Sound Health Care System, 1660 S Columbian Way, Seattle, WA 98108, USA; 8 Veteran Affairs Great Lakes Health Care System, VISN 12 PBM, 11301 W Cermak Road, Ste 810, Westchester, IL 60154, USA; karine.rozenberg@va.gov; 9 Division of Gastroenterology, Hepatology, and Nutrition, Virginia Commonwealth University, 1200 E Broad Street, West Hospital, 14th Floor, Box 980341, Richmond, VA 23298, USA; jasmohan.bajaj@vcuhealth.org; 10 Division of Gastroenterology, Central Virginia Veterans Affairs Healthcare System, 1201 Broad Rock Blvd, Richmond, VA 23249, USA; 11 Department of Medicine, Central Texas Veterans Healthcare System, 1901 Veterans Memorial Drive, Temple, TX 76504, USA; dawn.scott@va.gov; 12 VA Ann Arbor Healthcare System, 2215 Fuller Rd, Ann Arbor, MI 48105, USA; heather.mccurdy@va.gov; 13 Department of Infectious Diseases, Baltimore VA Medical Center, 10 N Greene Street, Baltimore, MD 21201, USA; emily.comstock@va.gov; 14 Salisbury VA Medical Center, 1601 Brenner Avenue, Salisbury, NC 28144, USA; michael.sidorovic@va.gov; 15 Digestive Disease Section, Cincinnati VA Medical Center, 3200 Vine Street, Cincinnati, OH 45220, USA; anna.nobbe@va.gov; 16 HIV, Hepatitis, and Related Conditions, Office of Specialty Care Services (10P11I), Department of Veterans Affairs, 810 Vermont Avenue, Washington, DC 20420, USA; maggie.chartier@va.gov (M.C.); david.ross4@va.gov (D.R.); 17 Gastroenterology Section, Veterans Affairs Puget Sound Health Care System, 1660 S Columbian Way, Seattle, WA 98108, USA; jason.dominitz@va.gov; 18 Division of Gastroenterology, Department of Medicine, RR-512, Health Sciences Building, University of Washington School of Medicine, Box 356420, 1959 NE Pacific Street, Seattle, WA 98195, USA; 19 Gastroenterology Section, VA Long Beach Healthcare System, 5901 E 7th Street, Long Beach, CA 90822, USA; timothy.morgan@va.gov; 20 Division of Gastroenterology, Department of Medicine, University of California, 333 City Blvd. West, Suite 400, Orange, CA 92868, USA

**Keywords:** implementation, hepatoma, learning collaborative, screening

## Abstract

**Simple Summary:**

Liver cancer is a growing problem that largely impacts people with cirrhosis. This article describes the Veterans Health Administration’s national cirrhosis quality improvement program and its focus on early detection of liver cancer.

**Abstract:**

After implementing a successful hepatitis C elimination program, the Veterans Health Administration’s (VHA) Hepatic Innovation Team (HIT) Collaborative pivoted to focus on improving cirrhosis care. This national program developed teams of providers across the country and engaged them in using systems redesign methods and population health approaches to improve care. The HIT Collaborative developed an Advanced Liver Disease (ALD) Dashboard to identify Veterans with cirrhosis who were due for surveillance for hepatocellular carcinoma (HCC) and other liver care, promoted the use of an HCC Clinical Reminder in the electronic health record, and provided training and networking opportunities. This evaluation aimed to describe the VHA’s approach to improving cirrhosis care and identify the facility factors and HIT activities associated with HCC surveillance rates, using a quasi-experimental design. Across all VHA facilities, as the HIT focused on cirrhosis between 2018–2019, HCC surveillance rates increased from 46% (IQR 37–53%) to 51% (IQR 42–60%, *p* < 0.001). The median HCC surveillance rate was 57% in facilities with high ALD Dashboard utilization compared with 45% in facilities with lower utilization (*p* < 0.001) and 58% in facilities using the HCC Clinical Reminder compared with 47% in facilities not using this tool (*p* < 0.001) in FY19. Increased use of the ALD Dashboard and adoption of the HCC Clinical Reminder were independently, significantly associated with HCC surveillance rates in multivariate models, controlling for other facility characteristics. In conclusion, the VHA’s HIT Collaborative is a national healthcare initiative associated with significant improvement in HCC surveillance rates.

## 1. Introduction

The incidence of hepatocellular carcinoma (HCC) continues to rise globally [1,2,3], paralleling the rising incidence of cirrhosis [4]. While guidelines recommend that persons with cirrhosis undergo biannual HCC surveillance with imaging [5,6,7,8], adherence to these guidelines remains low across all sectors of healthcare [9,10]. Such gaps between evidence or guidelines and clinical practice have been identified across healthcare systems and clinical areas. The Veterans Health Administration (VHA) is a national, learning healthcare system that has proactively addressed evidence to practice gaps and the so-called “quality chasm” by building capacity in systems redesign [11]. Adapted from manufacturing and engineering settings, systems redesign methods engage stakeholders in continuous quality improvement strategies to address the efficiency and safety of medical care [12].

VHA has been a national leader in teaching and using systems redesign methods to improve healthcare [13,14], particularly in the area of hepatitis C virus (HCV) elimination [15,16]. In 2014, VHA developed the Hepatic Innovation Team (HIT) Collaborative to implement systemic changes that would facilitate the roll-out of then-new direct-acting antiviral HCV treatments [16]. The HIT Collaborative remains a VHA-wide program of interdisciplinary healthcare providers organized into regional teams (defined by Veterans Integrated Service Networks or VISNs) and led by a national leadership team. This network of over 400 VHA clinicians and staff includes hepatologists, pharmacists, infectious disease providers, systems redesign experts, implementation scientists, and health services researchers. After working together to cure over 85% of Veterans with HCV, the HIT Collaborative transitioned to a focus on cirrhosis care in 2018. This transition leveraged the existing HIT infrastructure and leadership support to focus on cirrhosis care including HCC surveillance and surveillance for and treatment of varices [17,18,19].

This evaluation of the HIT Collaborative’s impact on HCC surveillance rates aimed to describe the VHA’s approach to improving cirrhosis care and identify the facility factors and HIT activities associated with HCC surveillance rates.

## 2. Materials and Methods

### 2.1. Cirrhosis and HCC Surveillance Definitions

The HIT Collaborative Leadership Team engaged a Technical Advisory Group of hepatologists, policy makers, researchers, and systems redesign experts to develop goals and metrics for cirrhosis care. Through a series of meetings, consensus-building activities, and review of the existing scientific literature, the data definitions of cirrhosis and HCC surveillance were established. Following national guidelines, 6-month surveillance was recommended, although an 8-month period was allowable for purposes of the measure, as recommended by the group.

All data were defined in and extracted from the VHA Corporate Data Warehouse and captured through a national VHA Advanced Liver Disease (ALD) Dashboard (described below). Veterans were defined as “in care” in their primary facility if they had at least one encounter or prescription over the prior 18 months. Veterans were associated with the VHA facility where they were assigned to primary care or, in the absence of primary care assignment, where they had their most recent visit or prescription activity in the prior 18 months. Veterans were classified as having cirrhosis based on diagnosis codes and problem list diagnoses consistent with cirrhosis or its complications (Supplementary Table S1: Cirrhosis codes and definitions). Veterans with cirrhosis were excluded from the HCC surveillance denominator for purposes of this evaluation if they had prior HCC or a limited life expectancy, defined as Child class C disease or a code for hospice care. Data sources used to define HCC surveillance included radiology codes, health factors specifying HCC surveillance, encounter and inpatient procedure codes, and fee-basis (non-VHA care) procedure codes. HCC surveillance was defined by the following CPT codes, which include abdominal ultrasound, MRI with contrast, or CT with contrast (74160, 74170, 74177, 74178, 74185, 74182, 74183, 76700, 76705). Imaging performed outside of VHA was also captured if it was manually input into the ALD Dashboard.

### 2.2. Facility Variables

Facility covariates that were assessed included complexity, specialty care availability, caseload, HIT participation, and adoption of HIT-related tools. Facility complexity was categorized following standard VHA nomenclature into five levels (1a, 1b, 1c, 2, and 3), using an algorithm that incorporates patient volume, patient morbidity, availability of services and specialties, and research resources, with level 1a representing the most complex facilities [20]. Facilities with on-site gastroenterology or hepatology specialists were considered to have “specialty care”. Facility caseload was defined as the number of Veterans with cirrhosis assigned to the facility at the end of fiscal year (FY) 2018, divided into quartiles.

### 2.3. HIT Membership and Use of the Advanced Liver Disease Dashboard, and HCC Clinical Reminder

HIT engagement was evaluated as (1) any participation at the facility, or any members of the facilities on the HIT rosters and (2) usage of HIT-endorsed data tools. The number of clinicians or staff members listed on the team roster was collected for each station, and engagement was defined as having at least one person on this team.

**Figure 1:**
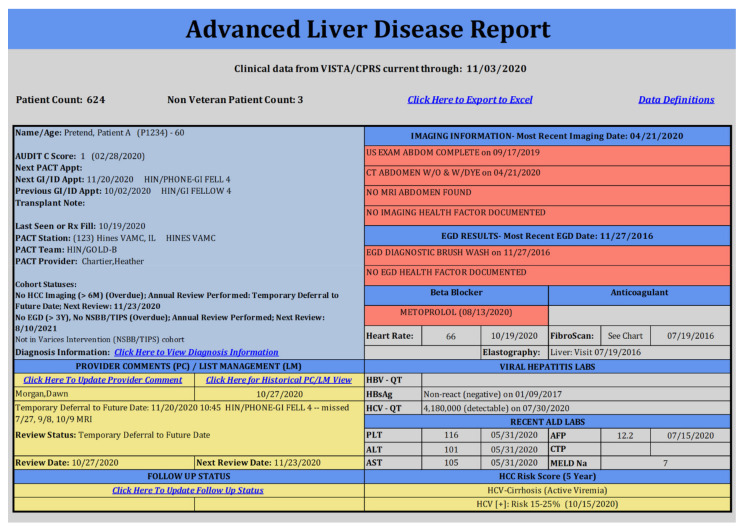
Mock Screenshot of Advanced Liver Disease Dashboard.

Two data tools were disseminated by the HIT: the Advanced Liver Disease (ALD) Dashboard and the HCC Clinical Reminder. The ALD Dashboard is a population management tool that was developed by a HIT-supported Dashboard Workgroup. Providers can use the ALD Dashboard to identify Veterans in their facilities and assess HCC surveillance status and other aspects of liver disease and overall health (Figure 1). Dashboard usage was categorized in two ways: (1) as a continuous number of logins and (2) as the continuous number of users, both at the facility level over the fiscal year.

The HCC Clinical Reminder activates in a Veteran’s electronic medical record when HCC surveillance is due. This HCC reminder was developed for national implementation by members of VISN 21 and offered to all facilities who opted in during FY18. If a facility had installed and turned on the HCC Clinical Reminder, the facility was defined as “using” the HCC Clinical Reminder in that fiscal year.

### 2.4. Analysis

All data analyses were conducted in RStudio version 1.2.5033. Figures and tables were created using Microsoft Office products (excel and word). Veterans were aggregated by facility and the median HCC surveillance rates at the facility level were compared at the end of FYs 2018 and 2019 using Wilcoxon rank sum testing. Multivariable linear regression models were used to assess the factors independently associated with HCC surveillance in each FY. In these models, ALD Dashboard use was modeled as high vs. low, defined by median number of logins, use of the HCC Clinical Reminder was included as a binary covariate, and caseload was included as a continuous covariate. Models were assessed for collinearity using a prespecified variance inflation factor of five.

## 3. Results

### 3.1. HIT Activities

The HIT Collaborative Leadership Team engaged regional teams in monthly calls and quarterly data reviews. These calls provided a forum for teams to share “best practices” and quality improvement work, using standard systems redesign tools [12,21]. The presentations and resources could be accessed on a SharePoint webpage between meetings. Additionally, the HIT Collaborative Leadership Team was available to provide technical assistance and coaching between calls. They provided education about the ALD Dashboard and HCC Clinical Reminder, trained clinicians in systems redesign methods, and developed a tool to estimate the cost of facility-level cirrhosis care. The Leadership Team offered on-site visits to all 18 VISN teams, during which time they worked with teams to set goals, assess data, and develop quality improvement strategies.

### 3.2. Facility Characteristics and HIT Engagement

At the start of 2018 (baseline), the majority of 130 VHA facilities had on-site specialty gastroenterology or hepatology care, and 87% had HIT members on site (Table 1). In FY18, 67,210 Veterans engaged in VHA care were identified as having cirrhosis, with this number increasing to 73,108 in FY19. In FY18, the median number of Veterans per facility with cirrhosis was 429 (IQR 238–689); this increased to 450 (264–773) in FY19. In FY18, there were 392 HIT members, with a median of two members per facility (range 1–15). In FY19, HIT membership grew to 476, with a median of three members per facility.

### 3.3. Advanced Liver Disease Dashboard

Between December 2017 and September 2018, the ALD Dashboard had been used by 125 of 130 facilities (96%) across all 18 VISNs. The total number of unique users was 562 (median four per site) and total number of logins was 3252 (median of 16 logins per user).

### 3.4. HCC Clinical Reminder

In FY18, at baseline, 21 facilities (16%) had adopted the HCC Clinical Reminder. By FY19, adoption doubled to 30%.

### 3.5. HCC Surveillance Rates

**Figure 2:**
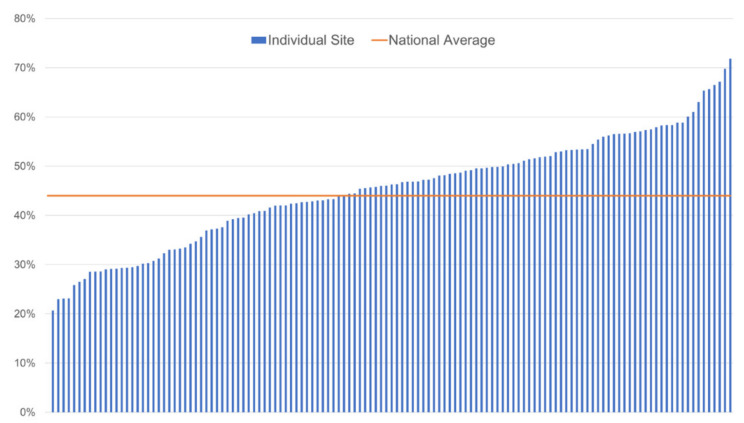
Proportion of Veterans receiving hepatocellular carcinoma surveillance in fiscal year 2018, by facility.

The median HCC surveillance rate in FY18 was 46% (IQR 37–53%) (Figure 2). Surveillance rates increased significantly to 51% (IQR 42–60%) by the end of FY19 (*p* < 0.01).

Several facility characteristics were associated with HCC surveillance rates in bivariate analyses (Table 2). Median HCC surveillance rates were 53% in facilities with gastroenterology or hepatology specialists on site compared with 41% in facilities without specialists, *p* = 0.003. Overall facility complexity was also significantly associated with HCC surveillance rates, with 49% surveillance in facilities with the highest complexity vs. 36% in those with lower complexity scores in FY18 and 54% vs. 44% in FY19 (*p* < 0.001).

Facilities with higher ALD Dashboard utilization and those utilizing the HCC Clinical Reminder had significantly higher HCC surveillance rates in both years (Table 2). The number of HIT members was not significantly associated with HCC surveillance in either year, but having at least one HIT member was significantly associated with HCC surveillance in FY19. In contrast, caseload was significantly associated with HCC surveillance rates only in FY18, when facilities with the higher caseloads had higher surveillance rates. However, over FY19, with HIT implementation, surveillance rates increased by a median of 8% in the facilities with the lowest vs. 1% in facilities with the highest caseload. Consequently, by the end of FY19, caseload was no longer significantly associated with HCC surveillance rate.

### 3.6. Factors Associated with HCC Surveillance in Multivariable Models

In multivariable models (Table 3), the factors independently, significantly associated with HCC surveillance in both years included ALD Dashboard utilization, HCC Clinical Reminder adoption, and higher facility complexity, controlling for other facility characteristics, and caseload (volume of Veterans with cirrhosis). Sensitivity analyses were conducted modeling HCC surveillance as an ordinal variable by quartile, with no significant changes in the associations.

## 4. Discussion

The HIT Collaborative is a novel, national VHA program that has employed a population health approach and systems redesign methods to address evidence to practice gaps in cirrhosis care. Over the first two years of the HIT’s focus on cirrhosis care, HCC surveillance rates significantly increased across a heterogenous group of facilities. The facilities with the most improvement were those that utilized the tools provided by the HIT, including an ALD Dashboard and an HCC Clinical Reminder. It is notable that smaller facilities without specialty care that used these tools were also able to increase their surveillance rates such that facility caseload and complexity were not associated with HCC surveillance rates once the HIT supports were in place.

Cirrhosis is a complex medical illness that requires ongoing chronic disease management. While prior studies have targeted specific aspects of cirrhosis care, such as prevention of readmission, this national, proactive, coordinated approach to surveillance is unique to VHA. Our evaluation demonstrated an association between a data-informed, provider-centered program and improved adherence to disease management guidelines.

While the HCC surveillance rates remained moderate, it is notable that the VHA was outperforming other healthcare systems in terms of HCC surveillance rates even prior to the HIT’s focus in this area. Guideline-concordant surveillance rates in other healthcare systems have been reported to be as low as 2–11%, with pooled estimate of HCC surveillance rates of 24% in a recent metanalysis [22,23,24]. VHA’s rate thus appears to be twice that of other healthcare systems and the continuous data monitoring and ongoing process improvement will aim to improve upon these rates. These findings indicate that the multipronged, proactive, population based VHA approach could benefit other integrated healthcare systems and may be adaptable to other healthcare settings.

Creating effective provider-facing interventions can be challenging, as there are often numerous barriers to implementing evidence-based medical practices [25]. It is generally appreciated that provider education and guideline dissemination are not sufficient to change practice [25,26]. In contrast, learning collaboratives are evidence-based implementation approaches that have had success in other clinical areas [27,28,29]. Learning collaboratives operate via building stakeholder interrelationships and employing modeling, education, and support to promote behavior changes. The HIT Collaborative specifically engaged stakeholders, provided a centralized data infrastructure with accompanying metrics and goals, and developed coordinated training and support for regional coordinators. The HIT Collaborative provided an infrastructure for communication and stakeholder engagement. Such an approach allowed for rapid dissemination of information and support for clinicians.

These data support that one key to VHA’s success in addressing evidence to practice gaps has been the development and wide adoption of data tools. Other research has demonstrated the effectiveness of data feedback both through audit-and-feedback mechanisms and through specific use of tailored electronic health record tools [30,31,32,33]. Notably, the combination of health record tools and systems redesign approaches have worked in other clinical areas [34]. However, none of these approaches are effective if they are not acceptable to clinicians and utilized in practice. The doubling of the HCC Clinical Reminder utilization in the first year of HIT implementation suggests that the HIT Collaborative helped to disseminate this clinical tool.

The ALD Dashboard provided information to clinicians and to leadership and was used to inform practice and policy. These data allowed national-level policymakers to evaluate efforts and develop tailored approaches to address implementation barriers. For example, in response to feedback, the Dashboard was adapted to allow providers to feedback data about why Veterans may not be engaged in surveillance (e.g., competing comorbidities, refusal). As we move forward, these data will allow the HIT Collaborative Leadership Team to design tailored strategies to address causes of non-surveillance. For example, if a primary barrier is related to refusal by Veterans to undergo surveillance, then direct patient outreach, transportation assistance, or bundled appointments could be employed to increase surveillance. In contrast, if scheduling or radiology capacity emerge as issues, then leadership can address this through internal, systems redesign approaches or engaging scheduling or radiology experts in quality improvement efforts. Thus, the bidirectional flow of information supports clinicians and leadership to improve the quality of care.

In any implementation effort, a one-size-fits-all approach is often insufficient. The HIT Collaborative has also allowed leadership to identify facilities requiring enhanced support. Ongoing work includes outreach to these facilities with opportunities to improve cirrhosis care [35]. This population-level approach allows leadership to target resources efficiently, providing more intensive resources to later adopting facilities. Such an approach is particularly beneficial when the system experiences stress, such as during the COVID pandemic.

## 5. Conclusions

VHA developed a national program that successfully focused on improving HCC surveillance rates for Veterans. By providing user-friendly data in the form of a Dashboard, clinical reminder, education, technical assistance, and an infrastructure for sharing best practices, VHA developed a population health approach to cirrhosis care that could be adopted by other healthcare systems aiming to improve access to evidence-based care.

## Figures and Tables

**Table 1:** Baseline Facility Characteristics (*n* = 130) *.

Characteristics	Facilities *n* (%)
Facility complexity	
1 (high)	77 (59%)
2	24 (18%)
3 (low)	29 (22%)
On-site GI specialty care	
Yes	106 (82%)
No	24 (18%)
HIT member at facility	
Yes	113 (87%)
No	17 (13%)
ALD Dashboard utilization	
High	34 (26%)
Low	96 (74%)
HCC Clinical Reminder use	
Yes	20 (15%)
No	110 (85%)

Abbreviations: GI: gastroenterology, which includes hepatology; HIT: Hepatic Innovation Team; ALD: advanced liver disease; HCC: hepatocellular carcinoma * high ALD Dashboard utilization = above the median number of log-ins.

**Table 2:** HCC surveillance rates in fiscal years 2018 and 2019 by facility characteristics.

Facility Characteristics	HCC Surveillance Rate (%)			
	FY18	*p*	FY19	*p*
Complexity		<0.001		<0.001
1 (high)	49		54	
2	44		47	
3 (low)	36		44	
Quartile of case load		<0.001		0.35
1 (low)	40		49	
2	43		50	
3	49		53	
4 (high)	49		51	
On-site GI specialty care		<0.001		0.003
Yes	48		53	
No	37		41	
HIT member at facility		0.12		0.003
Yes	41		52	
No	46		42	
ALD Dashboard utilization		<0.001		<0.001
High	51		57	
Low	40		45	
HCC Clinical Reminder use		<0.001		<0.001
Yes	53		58	
No	44		47	

Abbreviations: FY=fiscal year; GI: gastroenterology, which includes hepatology; HIT: Hepatic Innovation Team; ALD: advanced liver disease; HCC: hepatocellular carcinoma; high ALD Dashboard utilization = above the median number of log-ins.

**Table 3:** Multivariable linear regression models of HCC surveillance rates in fiscal years 2018 and 2019 *.

Covariates		FY18			FY19	
	β	SE	*p*	β	SE	*p*
Complexity	0.068	0.022	0.002	0.042	0.027	Ns
On-site GI specialty care	0.036	0.024	Ns	0.066	0.030	0.026
ALD Dashboard utilization	0.067	0.018	<0.001	0.072	0.022	0.001
HCC Clinical Reminder	0.054	0.025	0.029	0.076	0.023	0.001

* Caseload (number of Veterans with cirrhosis) and having a HIT member in the facility were included in the models but were not significantly associated with HCC surveillance in either year. Abbreviation: GI: gastroenterology; HIT: hepatic innovation team; ALD: advanced liver disease; Ns = non-significant (>0.05).

## Data Availability

These analyses were performed using VHA data. Deidentified data can be provided upon request pending ethical approval and in accordance with VHA guidelines and permissions.

## Supplementary Materials

The following are available online at https://www.mdpi.com/article/10.3390/cancers13092251/s1, Table S1: Cirrhosis codes and definitions.
