# Supplementary material for: The Hepatic Innovation Team Collaborative: A Successful Population-Based Approach to Hepatocellular Carcinoma Surveillance
Source: Cancers (Basel). 2021 May 7;13(9):2251. doi: 10.3390/cancers13092251 (PMC8125814; doi:10.3390/cancers13092251)
Supplement: Supplementary file 1 [file cancers-13-02251-s001.zip › cancers-1159384-supplementary.pdf]

cancers

MDPI

*Supplementary Materials*

# The Hepatic Innovation Team Collaborative: A Successful Population-Based Approach to Hepatocellular Carcinoma Surveillance

**Shari S. Rogal, Vera Yakovchenko, Rachel Gonzalez, Angela Park, Lauren A. Beste, Karine Rozenberg-Ben-Dror, Jasmohan S. Bajaj, Dawn Scott, Heather McCurdy, Emily Comstock, Michael Sidorovic, Sandra Gibson, Carolyn Lamorte, Anna Nobbe, Maggie Chartier, David Ross, Jason A. Dominitz and Timothy R. Morgan**

**Table S1.** Cirrhosis Codes and definitions.

| |
|---|
| (I85.00) esophageal varices without bleeding |
| (I85.01) esophageal varices with bleeding |
| (I85.10) secondary esophageal varices without bleeding |
| (I85.11) secondary esophageal varices with bleeding |
| (K65.2) spontaneous bacterial peritonitis |
| (K70.11) alcoholic hepatitis with ascites |
| (K70.30) alcoholic cirrhosis of liver without ascites |
| (K70.31) alcoholic cirrhosis of liver with ascites |
| (K70.40) alcoholic hepatic failure without coma |
| (K70.41) alcoholic hepatic failure with coma |
| (K71.51) toxic liver disease with chronic active hepatitis with ascites |
| (K71.7) toxic liver disease with fibrosis and cirrhosis of liver |
| (K72.10) chronic hepatic failure without coma |
| (K72.11) chronic hepatic failure with coma |
| (K72.90) hepatic failure, unspecified without coma |
| (K72.91) hepatic failure, unspecified with coma |
| (K74.3) primary biliary cirrhosis |
| (K74.4) secondary biliary cirrhosis |
| (K74.5) biliary cirrhosis, unspecified |
| (K74.60) unspecified cirrhosis of liver |
| (K74.69) other cirrhosis of liver |
| (K76.6) portal hypertension |
| (K76.7) hepatorenal syndrome |
| (K76.81) hepatopulmonary syndrome |
| (456.0) esophageal varices with bleeding |
| (456.1) esophageal varices without mention of bleeding |
| (456.20) esophageal varices in diseases classified elsewhere, with bleeding |

| |
|---|
| (456.21) esophageal varices in diseases classified elsewhere, without mention of bleeding |
| (567.23) spontaneous bacterial peritonitis |
| (571.2) alcoholic cirrhosis of liver |
| (571.5) cirrhosis of liver without mention of alcohol |
| (571.6) biliary cirrhosis |
| (572.2) hepatic encephalopathy |
| (572.3) portal hypertension |
| (572.4) hepatorenal syndrome |
| (103611000119102) Cirrhosis of liver due to hepatitis B ǀ disorder |
| (109819003) Obstructive biliary cirrhosis ǀ disorder |
| (123604002) Toxic cirrhosis ǀ disorder |
| (123605001) Nutritional cirrhosis ǀ disorder |
| (123606000) Cholangiolitic cirrhosis ǀ disorder |
| (12368000) Secondary biliary cirrhosis ǀ disorder |
| (123716002) Latent cirrhosis ǀ disorder |
| (123717006) Advanced cirrhosis ǀ disorder |
| (15999000) Mixed micro and macronodular cirrhosis ǀ disorder |
| (16070004) Syphilitic cirrhosis ǀ disorder |
| (1761006) Biliary cirrhosis ǀ disorder |
| (197279005) Cirrhosis and chronic liver disease ǀ disorder |
| (197291001) Unilobular portal cirrhosis ǀ disorder |
| (197293003) Diffuse nodular cirrhosis ǀ disorder |
| (197294009) Fatty portal cirrhosis ǀ disorder |
| (197296006) Capsular portal cirrhosis ǀ disorder |
| (197299004) Pigmentary portal cirrhosis ǀ disorder |
| (197301006) Toxic portal cirrhosis ǀ disorder |
| (197303009) Bacterial portal cirrhosis ǀ disorder |
| (197305002) Syphilitic portal cirrhosis ǀ disorder |
| (197310003) Biliary cirrhosis of children ǀ disorder |
| (197362001) Toxic liver disease with fibrosis and cirrhosis of liver ǀ disorder |
| (19943007) Cirrhosis of liver ǀ disorder |
| (21861000) Micronodular cirrhosis ǀ disorder |
| (235895002) Laennec's cirrhosis, non-alcoholic ǀ disorder |
| (235896001) Infectious cirrhosis ǀ disorder |
| (235897005) Hypoxia-associated cirrhosis ǀ disorder |
| (266468003) Cirrhosis - non-alcoholic ǀ disorder |
| (266469006) Multilobular portal cirrhosis ǀ disorder |
| (266470007) Cardiac portal cirrhosis ǀ disorder |

| |
|---|
| (266471006) Juvenile portal cirrhosis ǀ disorder |
| (271440004) Cirrhosis secondary to cholestasis ǀ disorder |
| (27156006) Posthepatitic cirrhosis ǀ disorder |
| (308129003) Esophageal varices in cirrhosis of the liver ǀ disorder |
| (309783001) Esophageal varices in alcoholic cirrhosis of the liver ǀ disorder |
| (33144001) Parasitic cirrhosis ǀ disorder |
| (371139006) Early cirrhosis ǀ disorder |
| (37688005) Clonorchiasis with biliary cirrhosis ǀ disorder |
| (399126000) Bronze cirrhosis ǀ disorder |
| (419728003) Portal cirrhosis ǀ disorder |
| (420054005) Alcoholic cirrhosis ǀ disorder |
| (425413006) Drug-induced cirrhosis of liver ǀ disorder |
| (43904005) Macronodular cirrhosis ǀ disorder |
| (45256007) Cruveilhier-Baumgarten syndrome ǀ disorder |
| (536002) Glissonian cirrhosis ǀ disorder |
| (6183001) Indian childhood cirrhosis ǀ disorder |
| (699189004) North American Indian childhood cirrhosis ǀ disorder |
| (715401008) Primary biliary cirrhosis co-occurrent with systemic scleroderma ǀ disorder |
| (716203000) Decompensated cirrhosis of liver ǀ disorder |
| (725416005) Cardiomyopathy co-occurrent and due to cirrhosis of liver ǀ disorder |
| (725938001) Cirrhosis of liver caused by methotrexate ǀ disorder |
| (725939009) Cirrhosis of liver caused by amiodarone ǀ disorder |
| (725940006) Cirrhosis of liver caused by methyldopa ǀ disorder |
| (735733008) Cirrhosis of liver co-occurrent and due to primary sclerosing cholangitis ǀ disorder |
| (74669004) Cardiac cirrhosis ǀ disorder |
| (76301009) Florid cirrhosis ǀ disorder |
| (78208005) Pigment cirrhosis ǀ disorder |
| (831000119103) Cirrhosis of liver due to chronic hepatitis C ǀ disorder |
| (86454000) Postnecrotic cirrhosis ǀ disorder |
| (89580002) Cryptogenic cirrhosis ǀ disorder |
